# Achromobacter xylosoxidans Purulent Bronchitis in a Previously Healthy Child: An Unexpected Consequence of COVID-19 Infection

**DOI:** 10.7759/cureus.21711

**Published:** 2022-01-29

**Authors:** Nusa Matijasic, Ana Tripalo Batos, Jasna Lenicek Krleza, Marijana Rogulj, Ivan Pavic

**Affiliations:** 1 Department of Pediatric Oncology and Hematology, Children's Hospital Zagreb, Zagreb, HRV; 2 Department of Pediatric Radiology, Children's Hospital Zagreb, Zagreb, HRV; 3 Department of Laboratory Diagnostics, Children's Hospital Zagreb, Zagreb, HRV; 4 Department of Pediatrics, University Hospital Centre Split, Split, HRV; 5 Department of Pediatrics, Children's Hospital Zagreb, Zagreb, HRV; 6 School of Medicine, University of Split, Split, HRV

**Keywords:** bronchitis, purulent, covid-19, xylosoxidans, achromobacter

## Abstract

*Achromobacter xylosoxidans *is an aerobic, Gram-negative rod with a broad intrinsic and acquired antimicrobial resistance, usually isolated in patients with cystic fibrosis (CF), immunodeficiencies, or those undergoing invasive procedures. We report a case of a previously healthy 14-year-old girl who was hospitalized in our institution due to a prolonged, progressive cough and exertional dyspnea, which started after a mild viral respiratory tract infection. To elucidate the cause of her symptoms, a bronchoscopy was finally performed, showing bilateral purulent bronchitis caused by *A. xylosoxidans*, isolated from bronchoalveolar lavage (BAL) sample. Since the patient had positive serological testing for coronavirus disease 2019 (COVID-19), we concluded that it was the initial viral infection, although of a mild clinical course, the one that created favorable conditions for proliferation and further inflammation caused by *A. xylosoxidans*.

## Introduction

*Achromobacter xylosoxidans* is an aerobic, Gram-negative rod with a broad intrinsic and acquired antimicrobial resistance. It is widely spread in aqueous environments but is also known to be a nosocomial colonizer. This emerging pathogen is becoming increasingly detected in respiratory specimens of adults and children with underlying lung conditions and/or immunodeficiency disorders, cystic fibrosis (CF) being the most commonly associated disease [1-7]. Recent data in pediatric CF patients show a strong correlation between *A. xylosoxidans* acquisition and faster lung function decline, more frequent hospitalizations, and the need for antibiotic courses [1]. The bacterium was first described in 1971 but it still remains underdiagnosed, unabling its early eradication. In addition, many cases are misidentified due to similarity with *Pseudomonas* species [2]. The diagnosis relies on isolation of the organism depending on the site of infection, primarily the respiratory tract, in which case the identification is made via culturing of bronchoalveolar lavage (BAL) fluid [3].

To our knowledge, there is only one described case of a serious *A. xylosoxidans* infection in a completely immunocompetent child, without any risk factors. Moreover, none of the cases of *A. xylosoxidans* infection have so far been associated with coronavirus disease 2019 (COVID-19) infection [2]. Therefore, we herein report a peculiar case of *A. xylosoxidans* purulent bronchitis in a previously healthy 14-year-old girl, following mild COVID-19 respiratory infection.

## Case presentation

A 14-year-old girl was hospitalized at the Department of Pediatrics, Children’s Hospital Zagreb, due to a prolonged productive cough and exertional dyspnea, lasting for four months. The symptoms had appeared during a convalescence period from a respiratory tract infection, which at the time clinically seemed viral. The girl was otherwise healthy, with an unremarkable medical history. Before admission, she had already undergone initial pulmonary workup in another hospital, revealing normal laboratory tests and chest X-ray, while interferon-gamma release assay excluded *Mycobacterium tuberculosis* infection as the probable cause of respiratory symptoms. Spirometry, however, verified a combined restrictive and obstructive pulmonary disorder with negative bronchodilator testing. Extensive allergology testing including immunoglobulin E (IgE) value, eosinophil cationic protein (ECP), nasal swab for eosinophils, exhaled nitric oxide level (FeNO), and skin prick test to inhalant allergens detected only a mild allergy to cypress.

**Figure 1:**
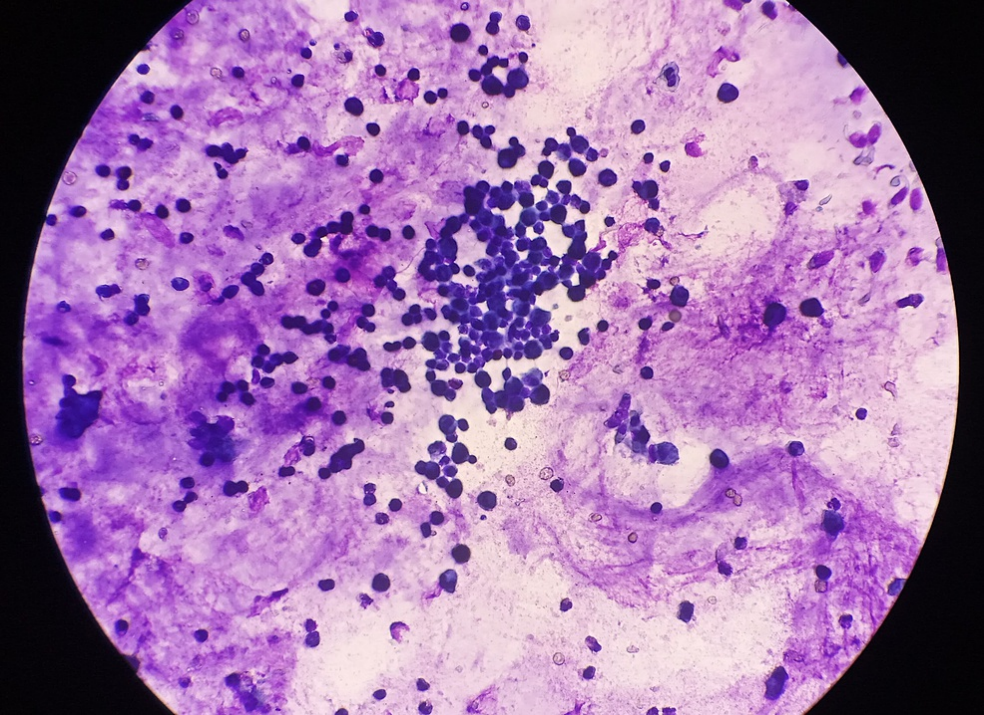
Microscopic view of the bronchoalveolar lavage sample showing mucus and inflammation mainly consisting of alveolar macrophages.

Upon admission to our clinic, the patient was in good general condition and afebrile, with inspiratory and expiratory coarse rales heard diffusely over both lungs. Hematological and biochemistry workup was all within the reference range, including inflammatory parameters. Polymerase chain reaction (PCR) testing for COVID-19 from nasal swab was negative. Control spirometry indicated a mild restrictive pulmonary disorder, while 24-hour impedance-pH monitoring did not detect laryngopharyngeal reflux. Echocardiogram and ultrasound of the heart excluded cardiac pathology. The patient was treated symptomatically with inhalations of isotonic and hypertonic saline and salbutamol, along with daily intensive respiratory therapy, unfortunately with minimal clinical improvement. To elucidate the etiology of the progressive productive cough and dyspnea, a bronchoscopy was performed, showing bilateral suppurative inflammation of the lower lung lobes. Macroscopically, the BAL sample was described as thick, yellow mucus. Finally, *A. xylosoxidans*, isolated in the BAL sample, was defined as the causative pathogen. Gram stain of BAL sample showed > 25 polymorphonuclear leukocytes per optic field, while cytological examination of the sample revealed inflammation consisting mainly of alveolar macrophages (66%) and neutrophils (20%), with scarce lymphocytes, eosinophils, and monocytes (Figure 1).

**Figure 2:**
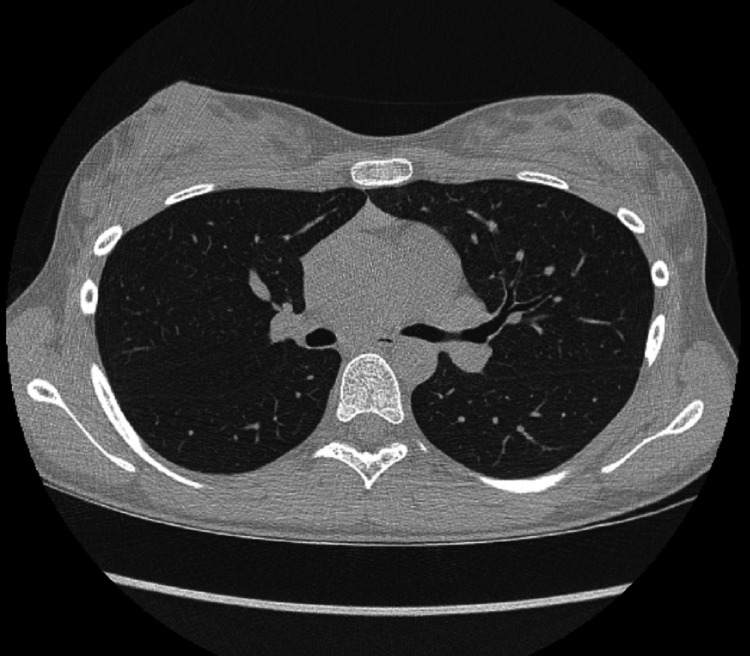
High-resolution computed tomography scan showing normal lung interstitium and parenchyma.

The finding was surprising, given that chronic lung disease and immunodeficiency, usually associated with *A. xylosoxidans* infection, were highly unlikely in our patient. Unfortunately, the bronchoscope suction valve and lavage fluid were not cultured, which could definitely rule out possible contamination. Screening for CF was negative, alpha antitrypsin level was normal, while high nasal nitric oxide measurements excluded ciliary dyskinesia. In addition, high-resolution computed tomography (HRCT) revealed a normal appearance of lung interstitium and parenchyma, without bronchiectasis (Figure 2).

In addition, subsequent broad immunological testing was negative for immunodeficiency disorders. Serological testing for COVID-19 was, however, positive, leading to the conclusion that the previous COVID-19 infection probably induced respiratory epithelium damage and local mucosal immunity dysfunction, causing infection with this rare bacterial pathogen.

According to the antibiogram, trimethoprim-sulfamethoxazole was introduced. Antibiotic therapy lasted three weeks and led to a major clinical improvement. Spirometry performed two months post-discharge verified normal lung function (Table 1).

**Table 1:** Spirometry findings before and after the treatment. VC - vital capacity; max - maximum; FVC - forced vital capacity; FEV - forced expiratory volume; MEF - maximal expiratory flow; PEF - peak expiratory flow.

Spirometry parameter	Before the treatment	After the treatment
VC max (%)	76	85
FVC (%)	76	83
FEV_1_ (%)	73	90
FEV_1_/VC max (%)	97	108
MEF_25% _(%)	63	92
MEF_50% _(%)	53	75
MEF_75%_ (%)	58	79
MEF_25%-75% _(%)	56	79
PEF (%)	63	82

## Discussion

*A. xylosoxidans* is an aerobic, Gram-negative, highly motile bacterium, usually detected in patients with CF, other underlying lung morbidities, immunodeficiency disorders, or in those undergoing invasive procedures. This opportunistic, ubiquitous pathogen can infect numerous organs but mainly attacks respiratory and urinary tracts. It has an affinity for aquatic surfaces. Due to its adaptability to survive in diverse environments, it was isolated in supposedly sterile hospital equipment such as respirators, humidifiers, ultrasound gels, etc. In a similar way to *Pseudomonas aeruginosa*, the bacterium possesses a denitrification system, enabling its survival and biofilm synthesis in hypoxic and anoxic airways of CF patients [1-7]. Other adaptive mechanisms leading to bacterium’s deleterious pathogenicity include hypermutability, microevolution, and subsequent ability to withstand fluctuating environmental conditions in CF lungs [6].

The prevalence of lung colonization in patients with CF nowadays ranges from approximately 10% to 18%, suggesting an increasing trend, in comparison to earlier reports [1,8-10]. According to a recent case-control retrospective study conducted in two major French pediatric CF centers, the acquisition of *A. xylosoxidans* was associated with accelerated lung function decline, leading to recurrent pulmonary exacerbations and hospitalizations of the affected children. The results were in accordance with studies obtained on adults with CF. However, the link between the presence of *A. xylosoxidans* and the decline of pulmonary function was difficult to establish, as children with positive isolates had worse lung function to start with. The hypothesis was that children with severe forms of CF were more prone to *A. xylosoxidans* infection, which further aggravated the disease course through cytokine production and chronic pulmonary inflammation [1].

In addition, being relatively rare and unrecognized until recently, *A. xylosoxidans* is often underdiagnosed and challenging to treat. Besides being intrinsically multidrug-resistant, the bacteria may also develop acquired resistance, both resulting from antibiotics’ efflux pumps and enzymatic degradation [6]. The most active antibiotics against the bacteria are piperacillin-tazobactam, meropenem, and trimethoprim-sulfamethoxazole. Treatment guidelines are still lacking, and treatment duration is not defined. Best results were reported to carbapenem therapy ranging from two to 14 weeks [1-3].

Contrary to the above-mentioned findings, our patient developed *A. xylosoxidans* purulent bronchitis, without underlying risk conditions. However, months preceding the first symptoms of purulent bronchitis, she suffered from a mild respiratory tract infection for which we hypothesized, based on a positive serological test, was COVID-19. This led us to think that possibly COVID-19 induced pulmonary epithelial damage and local immune disorder, enabling the acquisition and proliferation of *A. xylosoxidans*.

After angiotensin-converting enzyme 2 (ACE2)-mediated cell entry, COVID-19 induces a cytopathic effect, as a part of its replicative cycle. This activates cytokines release and recruitment of immune cells, which in most cases ensures recovery. In some patients, on the other hand, the virus can induce aberrant genes expression promoting a pro-inflammatory state, disrupted intercellular communication, and finally immune dysregulation, making the lung more vulnerable to secondary infections [11-14]. Other well-established negative viral effects on the respiratory tract include modulation of surface membrane receptors, inadequate mucociliary clearance, a decrease of chemotactic factors, increase of immature phagocytes, reduction of surfactant levels, and microbiome dysbiosis, all promoting bacterial superinfection [15].

## Conclusions

To our knowledge, this is the first reported case of *A. xylosoxidans *purulent bronchitis in a previously healthy child without underlying lung pathology or immunodeficiency, following COVID-19 infection. Secondary bacterial pneumonia and/or bronchitis caused by virally induced vulnerability of the respiratory tract and local immune dysregulation is known to be one of the leading causes of morbidity and mortality in COVID-19-affected patients. The shift to adaptive immune reaction toward viral infection fails innate immunity, which plays a significant role in bacterial protection via cytokines release and phagocytosis. This explains why bacterial superinfections usually occur as the virus is being eradicated from the lungs of COVID-19 patients. We, therefore, hypothesized that it was the COVID-19 infection, although of a mild clinical course, the one that created favorable conditions for proliferation and further inflammation caused by this rare bacterial pathogen.
